# Simple Staining of Cells on a Chip

**DOI:** 10.3390/bios12111013

**Published:** 2022-11-13

**Authors:** Fatma Betul Kosker, Omer Aydin, Kutay Icoz

**Affiliations:** 1 Department of Biomedical Engineering, Erciyes University, 38039 Kayseri, Türkiye; 2 Nanothera Lab, Drug Application and Research Center (ERFARMA), Erciyes University, 38039 Kayseri, Türkiye; 3 Department of Biomedical Engineering, Pamukkale University, 20160 Denizli, Türkiye; 4 Clinical Engineering Research and Implementation Center (ERKAM), Erciyes University, 38030 Kayseri, Türkiye; 5 Nanotechnology Research and Application Center (ERNAM), Erciyes University, 38039 Kayseri, Türkiye; 6 Department of Electrical and Electronics Engineering, Abdullah Gül University, 38080 Kayseri, Türkiye

**Keywords:** cell staining, passive microfluidics, immunomagnetic beads, colour signal, human eukaryotic cells

## Abstract

Simple staining of cells is a widely used method in basic medical diagnostics, education, and research laboratories. The stains are low-cost, but the extensive consumption results in excessive toxic waste generation. Thus, to decrease the amount of toxic waste resulting from the cell staining procedure is a need. In this study, we developed a magnetically driven and compartmentalized passive microfluidic chip to perform simple staining of human eukaryotic cells, K562 cells, and lymphocyte cells derived from patients. We demonstrated simple staining on cells with trypan blue, methylene blue, crystal violet, and safranin for high, medium, and low cell densities. The stained cells were imaged using a bright field optical microscope and a cell phone to count cells on the focal plane. The staining improved the color signal of the cell by 25-135-pixel intensity changes for the microscopic images. The validity of the protocol was determined using Jurkat and MDA-MB-231 cell lines as negative controls. In order to demonstrate the practicality of the system, lymphocyte cells derived from human blood samples were stained with trypan blue. The color intensity changes in the first and last compartments were analyzed to evaluate the performance of the chip. The developed method is ultra-low cost, significantly reduces the waste generated, and can be integrated with mobile imaging devices in terms of portability. By combining microfabrication technology with cell staining, this study reported a novel contribution to the field of microfluidic biosensors. In the future, we expect to demonstrate the detection of pathogens using this method.

## 1. Introduction

Manual cell staining is the conventional, gold-standard method that is used to visualize and investigate cells under a light microscope. The simple stain is a solution consisting of chromogen, which is a coloring molecule (often a benzene derivative), and a solvent (usually water or ethanol). The chromophore is the compound of the chromogen that imparts its color. Auxochromes are charged parts of chromogens that function as dyes to adhere stains to cells via ionizing groups. Most cells, including mammalian and bacterial cells, have negative charges on their membranes, which are drawn to by positively charged basic stains by exchanging ions. As a result of this interaction, the cell is colored. Frequently used basic stains are trypan blue (TB), methylene blue (MB), crystal violet (CV), and safranin [1,2].

A staining process incorporates fixing cells on a microscope slide, milliliter volumes of a stain, and washing steps in a laboratory setting. The simple staining procedure is a major tool used in various laboratories such as pathology, cell biology, and microbiology laboratories, and it allows one to distinguish target cells from others, quantify the cells, and examine the morphology and the cell structure. Conventional cell staining methods include heat-fixing cells isolated from the medium (culture or sample) on a microscope slide, which adheres cells to the surface of the glass slide. Even the simple staining process is inexpensive and relatively easy, i.e., using one stain, the procedure consumes high volumes of reagents, increasing the waste and making it prone to contamination, and cells cannot be transferred for further analysis. In staining processes that contain two or more staining steps, the aforementioned disadvantages increase [3,4,5].

In a study comparing automated staining systems and manual staining on blood culture samples, it was reported that automated staining systems could replace the manual method [6]. However, the main disadvantage of these instruments is their high cost, which most of the standard laboratories cannot afford. Therefore, manual staining, which is relatively cost-effective, is still widely used despite being prone to operator errors and high-volume consumption of toxic chemicals. It is essential to develop alternative methods that are simple to implement, time-saving, and generate less waste when compared to the conventional staining method for both research and central health laboratories [7,8].

As a result of the developments in microfabrication technology, microfluidics has emerged [9], and various applications in chemical and biological fields have been demonstrated [10,11,12]. One of the main advantages of microfluidic systems over standard laboratory techniques is reducing the reagent consumption and the waste generated. Staining techniques were integrated with microfluidics to observe the viability of the cells and monitor their movement. Cells were stained with fluorescent dyes and monitored instantly on the chip for the success of the separation process on the chip [13,14]. In microchips used for the detection of bacteria and viruses, off-chip staining was performed to determine the detection success by using fluorescent dyes, such as Fluorescein isothiocyanate isomer I (FITC) and SYBR Green II [15,16]. The presence of living cells in microfluidic chips developed for monitoring drug toxicity on cells also were detected by immunofluorescence staining in the compartments or channels where the cells were located [17,18]. DNA dyes such as DAPI were used to monitor cell degradation after fixation on microfluidic chips [19]. However, fluorescent dyes are expensive and require a fluorescent microscope.

Passive microfluidic systems do not utilize external connecting tubes, elements, and pumps, unlike conventional microfluidic systems [20,21]. The absence of these external components allows easier operation and mobility. In passive microfluidics, the progress of the fluids in the channels is obtained through the internal dynamics of the system, i.e., the system is kept at a certain slope or under pressure [21,22]. In some passive chips, liquids are transferred to the system through access holes by pipetting, and the liquids remain constant in the system while target cells loaded with magnetic particles are moved using an external magnetic field [23,24,25].

**Figure 1:**
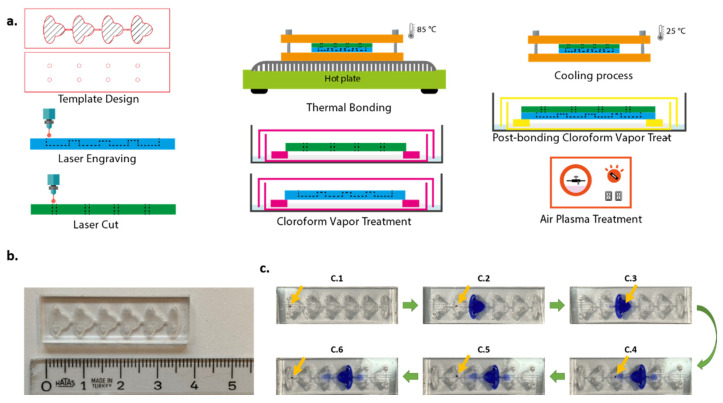
Chip fabrication procedure (**a**), upside view of the six-compartment chip (**b**). Staining procedure of magnetic bead-cell conjugates in the chip (**c**): (C.1–C.6) captured with mobile phone camera. Yellow arrows show the position of the cell–magnetic bead conjugates.

In this study, we proposed a passive microfluidic chip approach for performing cell simple staining procedures. We used magnetic particles to separate the target cells from a complex medium and then to move the cells in microfluidics by applying an external magnetic field. Either six (off-chip fixation) or eight (on-chip fixation) sequential compartments linked by microchannels made up of the microfluidic chip are shown in Figure 1. Each compartment features an inlet port for loading samples or reagents and an outlet port for ventilation. The microchannels connected the compartments and were located higher than the compartments’ base, and thus a capillary stop node was created. All the solutions, samples, and reagents were pipetted sequentially, one after the other, to the chip compartments. After being pipetted, solutions flow down the microchannel and become pinned at the capillary stop node. We first optimized the chip fabrication and tested the chip with immunomagnetically captured lymphoblast cells from the K562 cell line with different simple stains such as trypan blue, crystal violet, methylene blue, and safranin. Secondly, the immune cells derived from human blood samples were magnetically separated and added to the microfluidics for staining with trypan blue to demonstrate the feasibility of the system for patient samples. The magnetic field exerted on conjugates is found at an optimum level when seven cubic permanent magnets were used directly from the surface of the chip. The stained cells were inspected in the last compartment via bright field upright microscopes and a cell phone. Acquired images from the microscopes and the cell phone were analyzed using an in-house developed image processing algorithm. The method also enables taking stained cells out of the chip for further analysis, which is of high importance for genetic research of diseases. With the proposed method, a simple, low-cost, and easy-to-use cell staining system was obtained.

## 2. Experimental

### 2.1. Reagents and Materials

All solutions were prepared with phosphate-buffered saline (PBS, pH 7.4, Biological Industries, Beit Haemek, Israel). MACS buffer was prepared with 1% *w/v* bovine serum albumin (BSA, Sigma-Aldrich, St. Louis, MO, USA) and 2 mM ethylenediaminetetraacetic acid (EDTA, Sigma-Aldrich, St. Louis, MO, USA). Superparamagnetic beads conjugated with CD45 antibody (Dynabeads^®^ CD45, 4.5 µm diameter) were purchased from Invitrogen (Waltham, MA, USA). A fixation solution was prepared by dissolving 0.4 g paraformaldehyde powder (Sigma-Aldrich, St. Louis, MO, USA) in 10 mL PBS, previously heated to 50 °C. After BSA blocking with 0.5% BSA in PBS, the chip was washed with ultrapure water (18.2 MΩ CM at 25 °C).

The 25 cm × 25 cm × 1.5 mm Polymethylmethacrylate (PMMA) plates were purchased from McMaster Carr (Elmhurst, IL, USA), and 3 mm × 3 mm × 3 mm Neodymium N42 permanent cubic magnets (Hefei Super Electronics, Hefei, China) were purchased from a local hobby shop. Chip parts were fabricated using a laser system (Epilog Laser, Golden, CO, USA). For bonding chip parts, chloroform vapor (ZAG Kimya, İstanbul, Türkiye) was used. Chips were treated to air plasma with plasma cleaner (Harrick Plasma, Ithaca, NY, USA) for 1 min to improve hydrophilicity.

For staining, TB, 0.4%, was purchased from Sigma-Aldrich (St. Louis, MO, USA) and used at 0.1%, 0.05%, and 0.01% concentrations. CV, MB, and safranin were obtained from Norateks Kimya (İstanbul, Türkiye). CV and MB were diluted at a 1:10 ratio, and safranin was used directly without dilution.

### 2.2. Cell Culture

K562 acute myeloid leukemia cell line was obtained from American Type Culture Collection (ATCC). Cells were cultured in RPMI 1640 medium with 10% FBS addition at 37 °C with a steady flow of 5% CO_2_. Cultured cells were centrifuged at 500× *g* for 5 min and washed three times with PBS. In order to ascertain the number of viable cells, cells were stained with trypan blue (1:1) and counted manually using a Neubauer counting chamber (Sigma-Aldrich, St. Louis, MO, USA).

### 2.3. Patient Samples

Human lymphocyte cell samples were obtained from Genome and Stem Cell (GENKÖK) Research Center. The Erciyes University local ethics committee accepted the study protocol (#2021/178), and all patients provided written informed permission. Samples were taken from patients diagnosed with leukocyte adhesion deficiency (LAD). Samples used during the experiments were lymphocyte cells except for CD4+ T lymphocyte cells, i.e., they were negatively selected. By using the protocol included in the MojoSort^TM^ Isolation Kit, T cells were isolated (Biolegend, San Diego, CA, USA). Peripheral blood mononuclear cells (PBMCs), which are white blood cells, were collected from blood samples. After that, PBMCs were reconstituted in MojoSort^TM^ buffer, assessed, and optimized to 1 × 10^8^ cells per milliliter. Next, 10 mL of a biotin-antibody cocktail and 100:l of a cell suspension (10^7^ cells) were introduced to a second tube. A total of 10:l of the beads were vortexed into the mixture and allowed to incubate on ice for 15 min. After adding the sorting buffer, the sample was put in the magnet for five minutes before the liquid was collected. Sorting buffer was once again added to unlabelled fractions to isolate more CD4+ T cells from other CD4+ T cell subgroups.

### 2.4. Preparation of Cell–Magnetic Bead Conjugates

Cell pellet, ~3 × 10^6^ cells, after centrifugation, was suspended in 980 µL MACS buffer and mixed with 20 µL of superparamagnetic beads conjugated with CD45 antibody. The mixture was then gently incubated for 15 min at 4 °C via rotator (MACSmix Tube Rotator Sample Mixing, Miltenyi Biotec, Bergisch Gladbach, Germany). The cell–superparamagnetic bead conjugates were washed three times with sterile PBS using a magnetic separation rack (New England BioLabs, Hitchin, UK) and resuspended in 1 mL PBS buffer, 20 µL of which was then pipetted immediately to the first compartment of the chip.

### 2.5. Chip Fabrication

The chip design originated from [23]. Chip templates were designed using CorelDraw X7. The geometry and scale of the microfluidic chip are shown in detail in Figure S1. The thickness of PMMA sheets used as substrates was 1.5 mm. Raster engraving and vector cut features of the laser cutter were used for fabrication. The resolution was set to 500 dpi throughout the whole process. For the bottom part of the chip, a PMMA sheet was engraved to constitute compartments and channels. Access holes with 1 mm diameter were cut on another plane sheet to form the upper part of the chip. The volume of each compartment was approximately 20 µL.

Prior to bonding, chloroform vapor treatment (CVT) was applied to the parts of the chip for 3 min. Chloroform is a solvent for PMMA. When exposed to chloroform vapor, surface rigidity and opaqueness resulting from the engraving process were decreased. Immediately after CVT, parts of the chip aligned and bonded. This process enables only weak bonding. In order to strengthen the bonding process and prevent leakage, the chip was squeezed between two aluminum plates (Thor Labs BA2/M Mounting Base, 2″ × 3″ × 3/8″ (50 mm × 75 mm × 10 mm)). Then, this chip-in-aluminum plates complex was placed onto a hot plate, previously heated to 85 °C, for 10 min. After the thermal bonding process, the complex was cooled down at room temperature, 25 °C, for 10 min, and the chip was removed from the complex. In order to reduce the opaqueness resulting from heating, the chip was retreated with chloroform vapor for 2 min. The chip fabrication process is illustrated in Figure 1. The chips were also tested with food dyes to verify the fluid distribution (Figure S2).

### 2.6. Surface Modification of the Chip

PMMA surfaces are naturally hydrophobic. In order to obtain hydrophilicity and thus to easily spread the liquids in compartments and channels, the chip was treated with non-thermal atmospheric pressure plasma using Plasma Cleaner for 1 min.

The binding of the cells to the chip surface was a significant problem. In order to prevent cells from sticking, Bovine Serum Albumin (BSA) solution was applied as a blocking agent. The interior surfaces of the chip were washed with 0.5% BSA solution. Prior to cell experiments, excess BSA molecules were removed by washing with sterile PBS.

#### 2.6.1. Cell Staining Procedure

**Figure 2:**
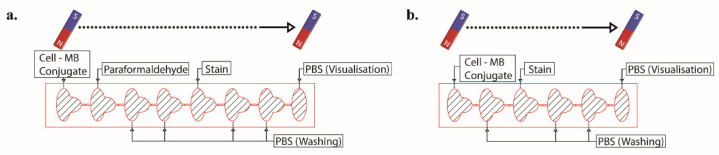
Schematic design of the chip with eight (**a**) and six (**b**) compartments for on-chip and off-chip configuration, respectively, and staining process showing solutions in compartments.

Cells were stained with either 0.1%, 0.05%, or 0.01% TB concentrations diluted from 0.4% TB and 1:10 dilutions of CV and MB stock solutions. Safranin was used directly from the stock. PBS buffer was utilized for dilution to discard stain aggregates. Some stains inherently have dark pigment coloring, i.e., trypan blue. In order to reveal the difference between the stained cells and the beads more clearly, we diluted the stain and obtained a lighter color. Dilution also helped to reduce the toxic effect of dyes on cells. All stain solutions were vortexed before use. A 4% paraformaldehyde solution was used to fix the cells. For on-chip fixation, the temperature of the paraformaldehyde solution was optimized and used at approximately 37 °C. The solution under this temperature caused lower fixed cells. For washing and visualization steps, sterile PBS buffer (pH 7.4) was used. All the solutions were pipetted sequentially, one after the other, to chip compartments using the inlet ports. Seven cubic neodymium magnets were facilitated to gather and transfer cells between adjacent compartments. All experiments were realized at room temperature, 25 °C. The schematic design of the chip and staining process is illustrated in Figure 2, and the workflow of the procedure is shown in Figure S3.

#### 2.6.2. Magnetic Force on Conjugate

**Figure 3:**
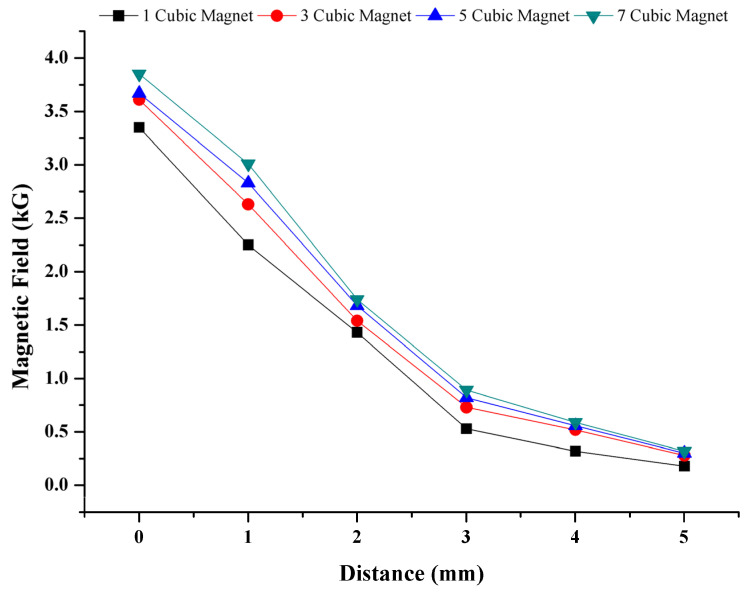
Magnetic field dependency on distance and number of magnets. Magnetic field on cell–magnetic bead conjugate is based on number of magnets and the distance from magnets.

In order to determine the magnetic force exerted on cell–magnetic bead conjugate, the magnetic field was measured by a digital gauss meter (FW 5180, FW Bell, Orlando, FL, USA). The 3 mm × 3 mm × 3 mm Neodymium N42 permanent cubic magnets (Hefei Super Electronics, China) were used to create the magnetic field. The magnetic fields generated by the different numbers of magnets from different distances were measured from the surface of the chip (Figure 3). As seen in the figure, the magnetic field weakens when the number of magnets is decreased, or the distance from the surface is increased. In order to provide a sufficient magnetic force on conjugates, seven cubic magnets directly from the surface of the chip were used during the experiments. When the distance between the magnets and the chip was above 0 mm, the time for gathering conjugates was longer, and the number of collected cells decreased. Optimization studies showed that further increasing the number of magnets was not necessary, which is consistent with the magnetic field vs. distance graphics in Figure 3. For thicker PMMA sheets, a higher number of magnets should be used to provide enough magnetic field.

In addition, one cubic magnet’s magnetic flux density was simulated in COMSOL Multiphysics software, the results of which can be seen in Figure S4. This simulation reveals that the magnet’s corner generates the highest magnetic field. Vertically stacked magnets were positioned at a right angle to the chip and moved forward during staining.

#### 2.6.3. Inspection and Analysis of Stained Cells in the Chip

Stained cells were inspected in the visualization compartment via bright field upright microscopes, Nikon Eclipse Ni with Nikon DS-Ri1 model CCD color camera using objectives 4×, 10×, 20× and Nikon Eclipse Ci (Nikon Instruments, Melville, NY, USA) with ToupCam XCAM1080PHA model CMOS camera using a 50× objective. After microscopic inspection, cells were transferred to a Neubauer counting chamber via pipette to determine the number of cells manually. In addition to microscope imaging, mobile phone imaging was also performed. Images were taken at 1.0×, 1.5×, and 2.5× magnifications with a 12 MP mobile phone camera (iPhone 11, Apple, Cupertino, CA, USA) under the light of a different mobile phone (Galaxy Note 4, Samsung, Seoul, South Korea) from approximately 10 cm from the chip.

Captured images from the bright field microscope and mobile phones were analyzed using the in-house developed algorithm in MATLAB programming language (R2021b, The MathWorks Inc., Natick, USA). The Colour intensity of stained cells and the number of cells were investigated.

## 3. Results and Discussion

### 3.1. Trypan Blue Concentration Experiments

Cell densities defined for the experiments were as follows: (i) high: number of cells > 50,000, (ii) medium: 30,000 < number of cells < 50,000, (iii) low: number of cells < 30,000. All procedures were realized at room temperature.

Cells counted and conjugated with superparamagnetic beads transferred to the first compartment of the eight-compartment-chip. As described in Figure 2, reagents for washing, PBS, and staining, TB, were introduced to the compartments sequentially. After all of the compartments were filled, cell–superparamagnetic bead conjugates were transported to the next compartment, first to the fixation compartment via permanent magnets. Conjugates remained here for 5 min for fixation. Fixed conjugates were moved to washing compartments and stayed in the compartments for 0.5 min. Conjugates were proceeded to the following compartment and stained with 0.1% TB solution for 5 min. After the staining step, conjugates were washed two times with PBS in the washing compartments and were moved to the last compartment for visualization.

The 0.1%, 0.05%, and 0.01% TB concentrations diluted from 0.4% TB stock solution were used to stain cells on the chip. Stained cells at the last compartment of the chip were visualized with a bright field upright microscope (4×, 10×, 20×, and 50× objectives), and then acquired images were analyzed in MATLAB.

Firstly, the region of interest was cropped from the whole image, cells in this region were selected, and red, green, and blue color intensity values were obtained. At least 20 cells were analyzed from various regions, and averages were calculated.

**Figure 4:**
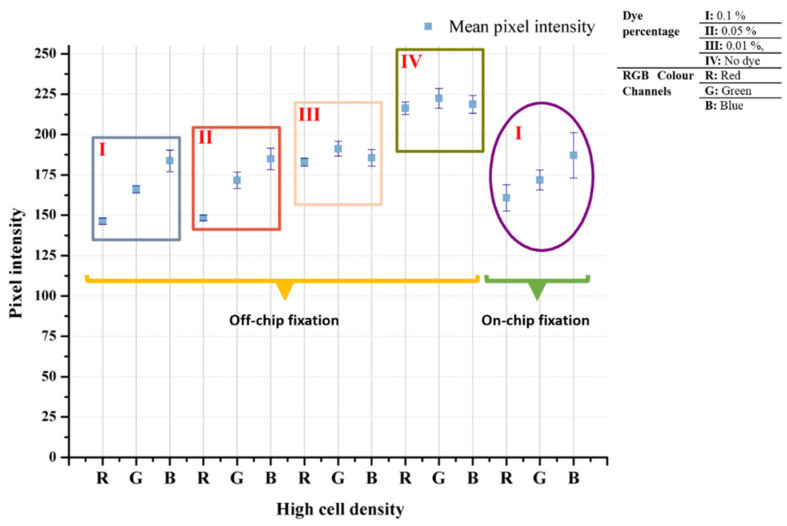
Trypan blue concentration effect on cell staining on off-chip and on-chip configuration. The 0.1%, 0.05%, and 0.01% TB dilutions from 0.4% stock solution were used on high number of cells.

As seen in Figure 4, color intensity values increased while the TB dilution factor increased from 0.1% to 0.01%. This is because as the color concentration decreases, each cell color uptake decreases, and the pixel intensity value rises. Although the change in the blue channel was small, it continued to increase as the concentration increased. According to the results of the analysis, the channel with the greatest change was the red color channel. When the change in both blue and green channels is considered together, it was observed that the color shifted to green tones as the concentration of the dye decreased.

### 3.2. Staining with Different Simple Stains

**Figure 5:**
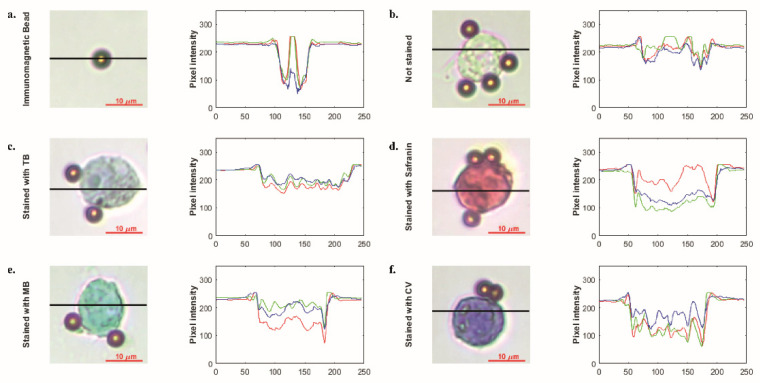
Microscopic images immunomagnetic bead (**a**) and single-cell unstained (**b**) and stained with simple stains such as trypan blue (**c**), safranin (**d**), methylene blue (**e**) and crystal violet (**f**), respectively. Graphics illustrate pixel intensity analysis results of related microscopic images. Images were acquired with 50× objective.

In order to show that the developed biochip can be used to stain cells with different stains, safranin, methylene blue, and crystal violet were pipetted into the staining compartment, and stained cells were imaged using the microscope some of which can be seen in Figure S9 The pixel intensity changes were examined to show the cell staining differences, i.e., bead vs. stained cell or unstained cell vs. stained cell. Average differences from the background are given in Table 1. The mean pixel intensity was calculated at 225 for the background. Even in the unstained cell, which was the closest to the background, a difference value of at least a 15-pixel intensity value was observed in each color band. Among the color bands, the dominant color of the dye has higher values than the other color bands, i.e., for safranin staining, red is the dominant color and has higher pixel intensity. Thus, the distinctive color bands are the bands other than the dominant color band. In this study, single- or two-stage thresholds were used to distinguish stained cells from others, beads, or the background. As seen in Figure 5, staining resulted in 25-135-pixel intensity changes for each color channel, which can be detected by image processing algorithms.

### 3.3. Control Experiments with Jurkat and MDA-MB-231 Cell Lines

**Figure 6:**
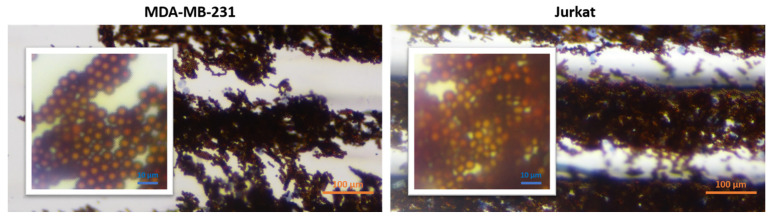
Last compartment microscopic images (10×) of Jurkat and MDA-MB-231 cell lines. Inset images show a close view of the original images.

In order to determine the validity of the protocol, Jurkat leukemic T cell and MDA-MB-231 breast cancer cell lines (obtained from ATCC), both of which are CD45 negative (CD45-), were used as the negative control. Cells were subjected to the same protocol as for K562 cells. As seen in Figure 6, no cells were observed in the last compartment. Only magnetic bead clusters were visible. In some trials, a very small number of cells, 1–10 cells, remained between the beads, which indicates nonspecific binding. Magnetic beads were moved to the staining compartments on the chip as in the normal procedure. Since there was no cell attachment, no staining was observed in the magnetic bead cluster in the last compartment. The last compartment images of both cell lines are given in Figure 6.

Cells were examined in flow cytometry (FC) to confirm that the cells were CD45-. FC results showed that our cells are not expressing CD45 cell receptor antigens. The results are given in Figures S7 and S8.

### 3.4. Mobile Phone Image Analysis

**Figure 7:**
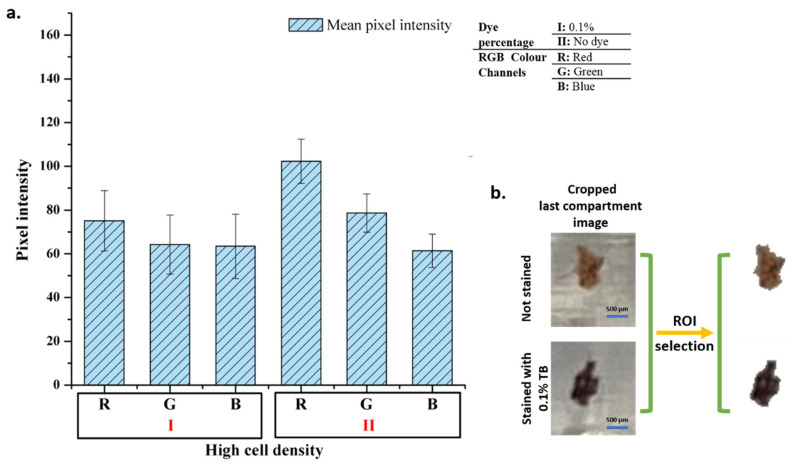
Mobile phone image analysis. Pixel intensity analysis results of the images (**a**) and image processing step (**b**).

In order to determine whether the biochip can be integrated with a simpler imaging system, mobile phone imaging was also performed. Images were taken at 1.0× magnification with a 12 MP mobile phone camera (iPhone 11, Apple) under the light of a different mobile phone (Galaxy Note 4, Samsung) from a distance of approximately 10 cm from the chip. The images were transferred to the computer and processed in the Matlab programming language. First, areas of the cell–magnetic bead complex were identified and separated from the rest of the image. The size of the area was determined, and the average pixel intensity values were found for each color band. The analysis results of the experiments with 0.1% TB and without dye taken at 1.0× magnification are given in Figure 7.

As in microscopic cell analysis results in Figure 4, the highest difference, at least 20% change, is seen in the red color band in mobile phone image analysis. Analyses show that there is no significant change in the blue color band. However, the change in red and green color channels is sufficient to distinguish stained cells from unstained cells. Therefore, the designed system can be used for basic cell staining differentiation.

### 3.5. Performance Analysis of the Chip

**Figure 8:**
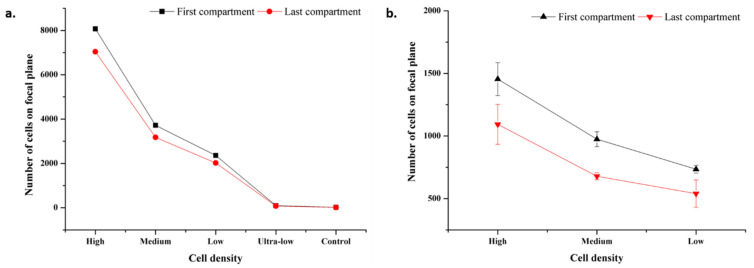
Microscopic and mobile images focal plane analysis results. (**a**) Microscopic image analysis results for cell densities at high, medium, low, ultra-low cell, and control. Standard deviation bars are lower than 15. As scale increment value is 1000, standard deviation bars cannot be seen. (**b**) Mobile image analysis results for cell densities at high, medium, and low.

Different cell densities were analyzed based on the areas occupied by the cell–magnetic bead complex on both microscopic and mobile phone images. For microscopic images, analysis was performed on microscope images taken with a 4× objective. First, the area occupied by the conjugates and an average cell was determined from the image. A value was obtained for each cell density by the ratio of the area occupied by the conjugates to the total cell area. This value was calculated for both the first compartment and the last compartment. The graph of the analysis is given in Figure 8a. The first and last compartment values’ range is 8070-95 and 7036-77 from high to ultra-low cell densities, respectively. Results follow a consistent trend downwards or vice versa. Conjugate transport efficiency and conjugate losses were also calculated from the data (See Section S5 and Figure S5). Conjugate transport efficiency was obtained as more than 80% for each cell density which relates to 20% conjugate loss at most.

For mobile phone images, analysis was performed on images captured at 1.0× magnification for high, medium, and low cell densities. The same procedure was followed as in microscopic image analysis. The values vary in the range of 1455-735 and 1093-540 from high to low cell densities, respectively. The analysis results are shown in Figure 8b. The standard deviation is high compared to microscopic image values, which is a result of device resolution.

### 3.6. Patient Samples’ Experiments

Cell analyses, for example, genetic analysis, constitutes a significant place in the research of diseases. Therefore, it is essential to store and process the samples taken from a living subject to utilize them at maximum efficiency. In this study, human lymphocyte cells were stained with 0.1% TB. In future studies, it will be possible to take the stained cells from the last compartment of the chip and carry out cell analysis.

**Figure 9:**
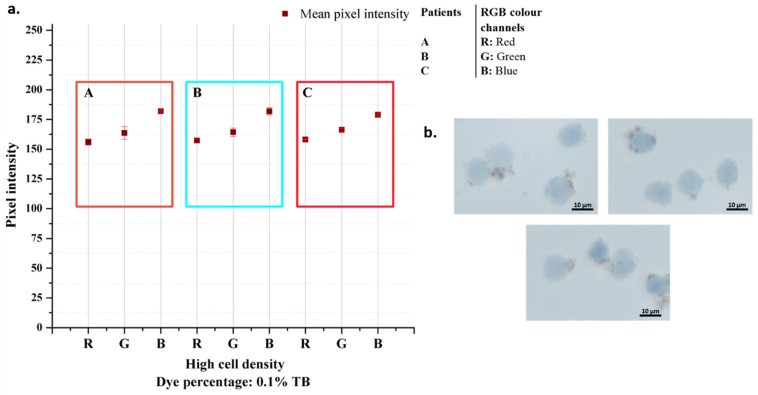
Patient samples analysis results (**a**) and their microscopic images taken with 50× objective (**b**).

**Figure 10:**
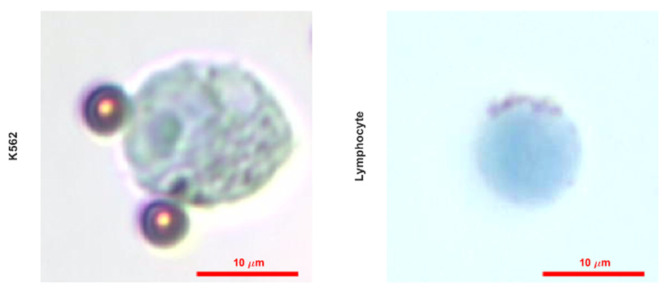
Microscopic images of single K562 and lymphocyte cells stained with 0.1% TB taken with 50× objective.

After the cells were taken, they were fixed, and the standard procedure was continued. Since the cells are smaller than the K562 cells, the cell density used in the experiment was determined on a larger scale. The images obtained as a result of the experiments and the graphic in which the data were analyzed are presented in Figure 9. The color analysis results for all three samples were observed consistently for each color band, and the standard deviation was quite low. The fact that the cells are smaller than K562 cells, approximately 2.5 times, and the magnetic beads are nano-sized is the main reason for the consistent results (Figure 10). It should be considered that there are differences in the staining percentages and their reflections on the color values of cells taken directly from the body without any cell culture treatment.

In this study, it was possible to take out the stained cells from the outlet port and then examine them for further analysis. In addition, by cutting off from the air contact of the inlet and outlet ports of the chip, i.e., covering the chip with parafilm, the stained cells can stay in a humid and closed environment at +4 °C for about 3 months.

The advantages of magnetic particles were demonstrated in various reports [26,27]; in this study, they were used for magnetic separation and movement of cells in microfluidics. When the magnetic bead size is considered, it is obviously seen that the images of nano-sized magnetic bead conjugated cells are explicit, and the accumulation of beads is avoided.

The usage of high volumes of reagents is one of the main drawbacks of the conventional cell staining process, which increase the cost and produce toxic waste (toxigenic stains). Our low-cost microfluidic device addresses these issues by lowering the reagents’ volume, especially stains, down to 5 µL per compartment; hence low toxic waste is generated. A comparison of the developed method with the related methods in the literature is shown in Table 2.

Micro/nano technology-based designs aim to analyze small volumes, i.e., a drop, of human samples such as blood, saliva, or urine via portable systems. Therefore, analyzing small sample volumes thoroughly without losing any information is important. In this manner, the presented method provides retrieval of the stained cells even after 3 months for further analyses.

The current sensitivity of the system is presented in Figure S6, which shows a linear range between 5000 and 50,000 input cells. The system is able to detect a minimum of ~77 cells in the focal plane. After improving the design, all the stained cells can be dispersed to a single focal plane, and then the staining chip can be used to quantify target cells.

In future studies, it is planned to use a simple handheld microscope for the design of a portable system. When the advantages of the system designed in the study, i.e., simplicity, low-cost, and low-waste generation, combined with the portable system, on-site inspection will be available.

## 4. Conclusions

In this study, a passive microfluidic device for rapid, accurate, and cost-effective cell staining procedures was demonstrated. Human eukaryotic cells, K562 cells, and lymphocytes from patients were stained with the developed chip. The stained cells were visualized by a bright field optical microscope and a cell phone. Staining resulted in 25–135-pixel intensity changes for each color channel for microscopic images, which was detected by an in-house developed algorithm. Cell phone images were also analyzed, and the change in red and green color channels was found to distinguish the stained cells from the unstained cells. When compared, nano-sized magnetic beads were found to provide more consistent results. In order to analyze the performance of the chip, the number of cells on the focal plane per cell density was calculated, and the results showed a trend. Conjugate transport efficiency was achieved by more than 80%, which relates to 20% conjugate loss at most per cell density. The fabrication of microfluidics does not involve any expensive cleanroom procedure or equipment as many micro systems require [28,29]. The developed method’s key benefits are simplicity and portability. Thus, the system does not require bulky off-chip equipment such as pumps. The method does not require any expensive fluorescent dyes for further analysis. In addition, the number of chemicals and sample volume needed throughout the experiment were significantly reduced. Only 5 μL of stain was used to color the cells. Minimum waste generation is another important advantage of the chip, so this system is an environment-friendly cell-staining. We demonstrated cell phone imaging to investigate the feasibility of the staining for a portable detection system.

The proposed method is also appropriate for staining and imaging of pathogenic and toxigenic cells due to its capped and closed structure, and thus, possible contamination will be prevented. We plan to further develop the system for the detection of cells and pathogens where a portable imaging system is integrated.

## Figures and Tables

**Table 1:** Difference values from background for bead, unstained cell, and cells stained with basic stains.

Difference from Background for:	Color Bands		
	R	G	B
Bead	125	120	140
Unstained cell	15	15	30
Cell stained with TB	50	40	40
Cell stained with Safranin	25	135	100
Cell stained with MB	100	100	45
Cell stained with CV	100	115	30

**Table 2:** Comparison of developed method with related staining methods in the literature.

Method	Volume	Price	Portability	Retrieval of Stained Cells	References
Standard staining	milliliters	>$5	No	No	[1,2,3,4,5]
Micro-staining	microliters	<$1	No	No	[8]
Microfluidics	microliters	10 cents	Yes	Yes	This study

## Supplementary Materials

The following supporting information can be downloaded at: https://www.mdpi.com/article/10.3390/bios12111013/s1, Figure S1: Microfluidic chip from different perspectives; Figure S2: Verification experiments for chip operation; Figure S3: Schematic overview of the staining procedure on the chip; Figure S4: Magnetic flux density simulated in COMSOL Multiphysics software; Figure S5: Microscopic image analysis results; Figure S6: Sensitivity analysis results; Figure S7: Flow cytometry analysis results for MDA-MB-231 cell line; Figure S8: Flow cytometry analysis results for Jurkat cell line; Figure S9: Microscopic images (50x) of K562 cells stained with different simple stains..
