# Supplementary material for: Simple Staining of Cells on a Chip
Source: Biosensors (Basel). 2022 Nov 13;12(11):1013. doi: 10.3390/bios12111013 (PMC9688635; doi:10.3390/bios12111013)
Supplement: Supplementary file 1 [file biosensors-12-01013-s001.zip › biosensors-1991860-supplementary.pdf]

biosensors

Simple Staining of Cells on a Chip

Fatma Betul Kosker [1,2,3], Omer Aydin [1,2,4,5,*] and Kutay Icoz [6,*]

1 Department of Biomedical Engineering, Erciyes University, 38039 Kayseri, Türkiye
2 Nanothera Lab, Drug Application and Research Center (ERFARMA), Erciyes University, 38039 Kayseri, Türkiye
3 Department of Biomedical Engineering, Pamukkale University, 20160 Denizli, Türkiye
4 Clinical Engineering Research and Implementation Center, (ERKAM), Erciyes University, 38030 Kayseri, Türkiye
5 Nanotechnology Research and Application Center (ERNAM), Erciyes University, 38039 Kayseri, Türkiye
6 Department of Electrical and Electronics Engineering, Abdullah Gül University, 38080 Kayseri, Türkiye
* Correspondence: omeraydin@erciyes.edu.tr (O.A.); kutay.icoz@agu.edu.tr (K.I.)

SUPPLEMENTARY INFORMATION

1. Close view of the chip

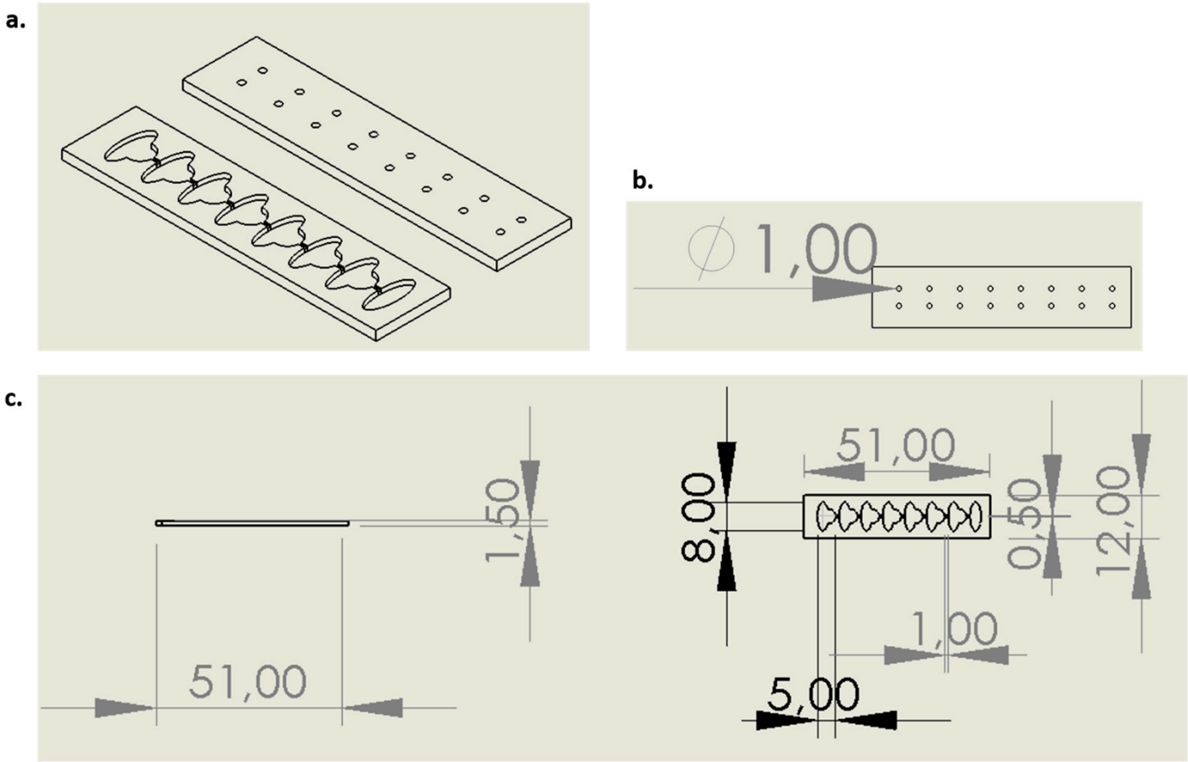

Figure S1. Microfluidic chip from different perspectives: (**a**) Isometric view of the upper and bottom part of the chip, (**b**) Top view of the upper part of the chip, (**c**) Side and top view of the bottom part of the chip.

2. Verification analysis of the chip

Before performing cell experiments, we also examined our chip with coloured (food dye) deionized water to verify the operation of the capillary valves. Some of the results are presented in Figure S2. After verification of our chip together with the information stated in references [23] and [25], we continued with the cell experiments.

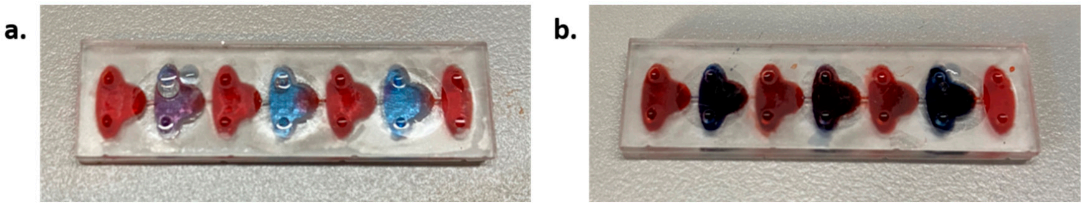

Figure S2. Verification experiments for chip operation.

3. Staining procedure on the chip

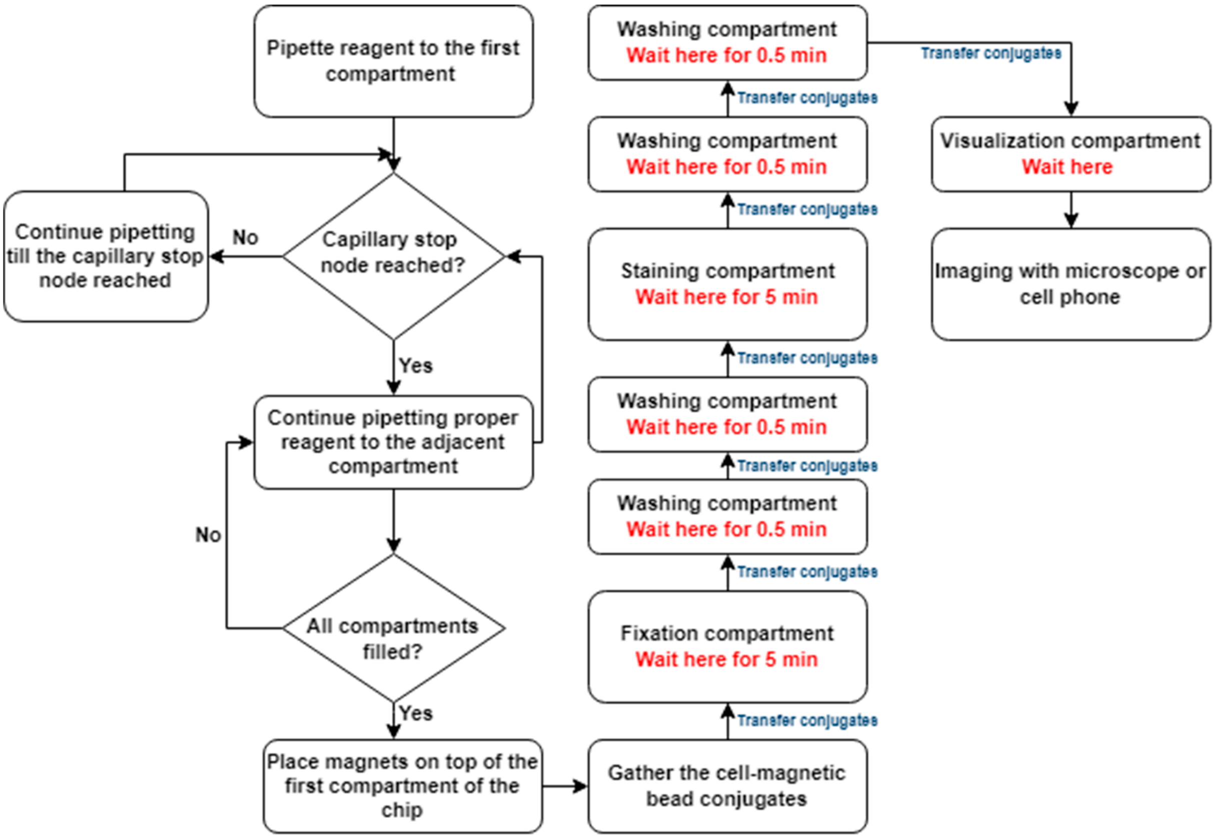

Figure S3. Schematic overview of the staining procedure on the chip.

4. Simulation of magnetic flux density

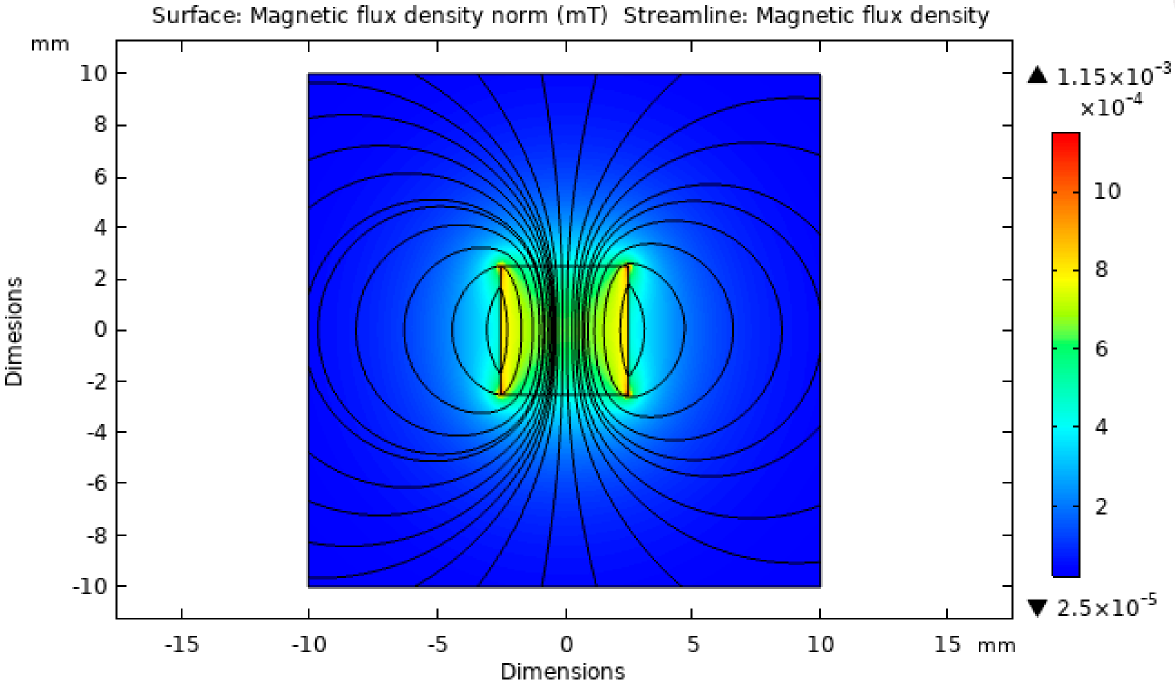

Figure S4. Magnetic flux density simulated in COMSOL Multiphysics software.

5. Performance analysis of the chip

The area occupied by the conjugates and an average cell was determined from microscope images taken with a 4x objective. A value was obtained for each cell density by the ratio of the area occupied by the conjugates to total cell area. This value was calculated for both the first compartment (FiC) and the last compartment (LaC). The cell transport efficiency and cell losses were determined from these values as given in equations 1 and 2. The graph of the data obtained from equations is given in Figure S2. As can be seen from the graph, the cell transportation efficiency value increased following a trend as the cell density increased. Cell transport efficiency was also obtained as more than 80% for each cell density.

$$cell\ transport\ efficiency = \frac{LaC\ value}{FiC\ value} \times 100 \quad (1)$$

$$cell\ loss = \frac{FiC\ value - LaC\ value}{FiC\ value} \times 100 \quad (2)$$

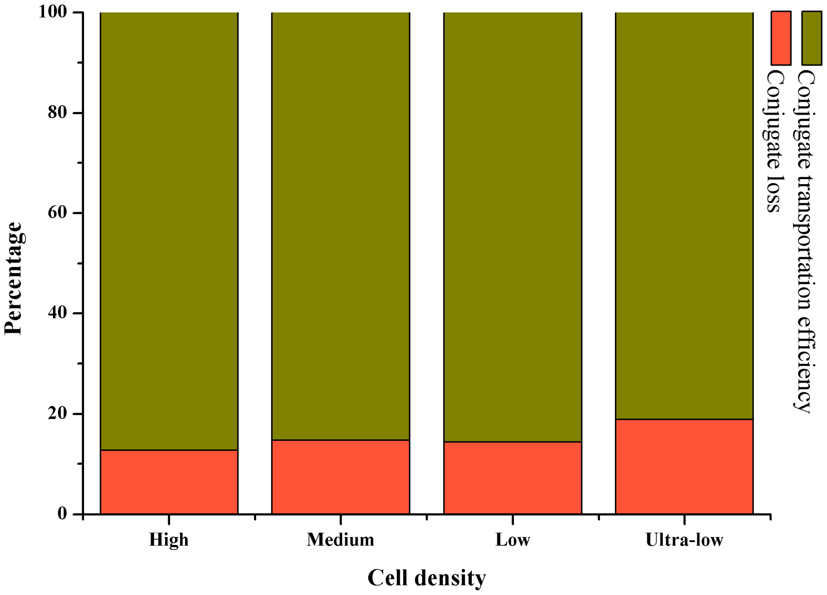

Figure S5. Microscopic image analysis results.

6. Sensitivity analysis of the chip

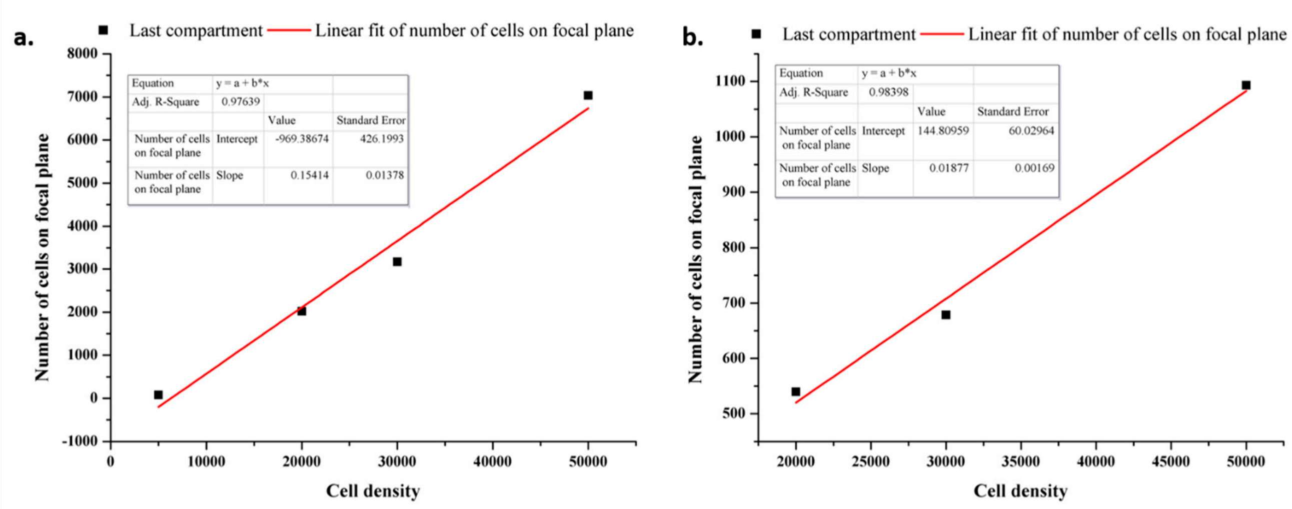

Figure S6. Sensitivity analysis results.

For sensitivity analysis, cell densities were attributed approximate numbers

7. Flow Cytometry Analysis of Jurkat and MDA-MB-231 cell lines

MDA-MB-231 and Jurkat cell line CD45 expression was investigated by using a flow cytometer (Guava easyCyte). Both cell lines were stained with Alexa Fluor 594 goat anti-rabbit secondary antibody purchased from Invitrogen (USA).

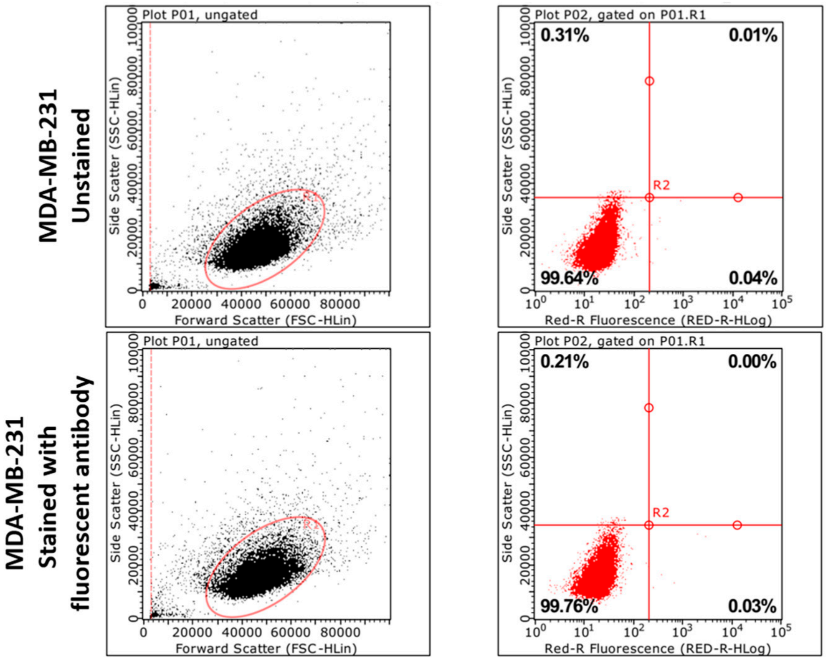

Figure S7. Flow cytometry analysis results for MDA-MB-231 cell line.

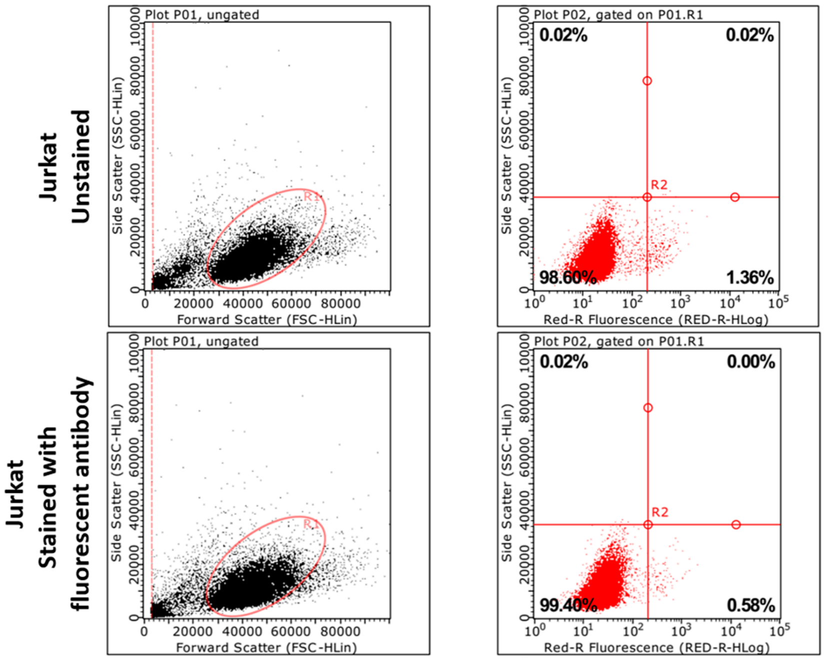

Figure S8. Flow cytometry analysis results for Jurkat cell line.

8. Staining with different simple stains

biosensors

MDPI

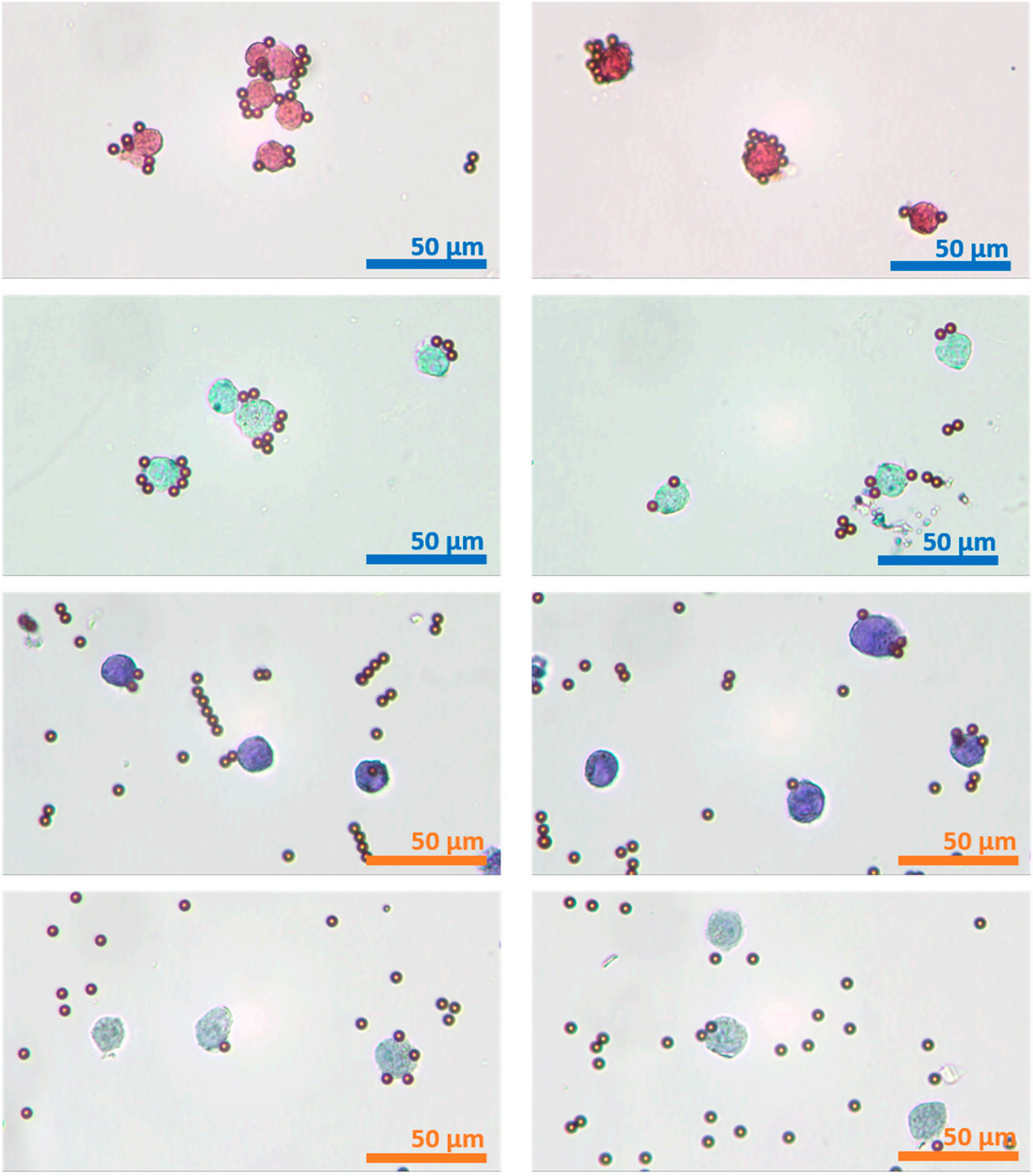

Figure S9. Microscopic images (50x) of K562 cells stained with different simple stains, safranin, methylene blue, crystal violet and trypan blue, respectively.
